# Supplementary material for: Transcriptional CDK Inhibitors as Potential Treatment Option for Testicular Germ Cell Tumors
Source: Cancers (Basel). 2022 Mar 26;14(7):1690. doi: 10.3390/cancers14071690 (PMC8997165; doi:10.3390/cancers14071690)
Supplement: Supplementary file 1 [file cancers-14-01690-s001.zip › cancers-1644300 supplementary/cancers-1644300 supplementary figures.pdf]

CDK inhibitors
-Supplementary Figures-

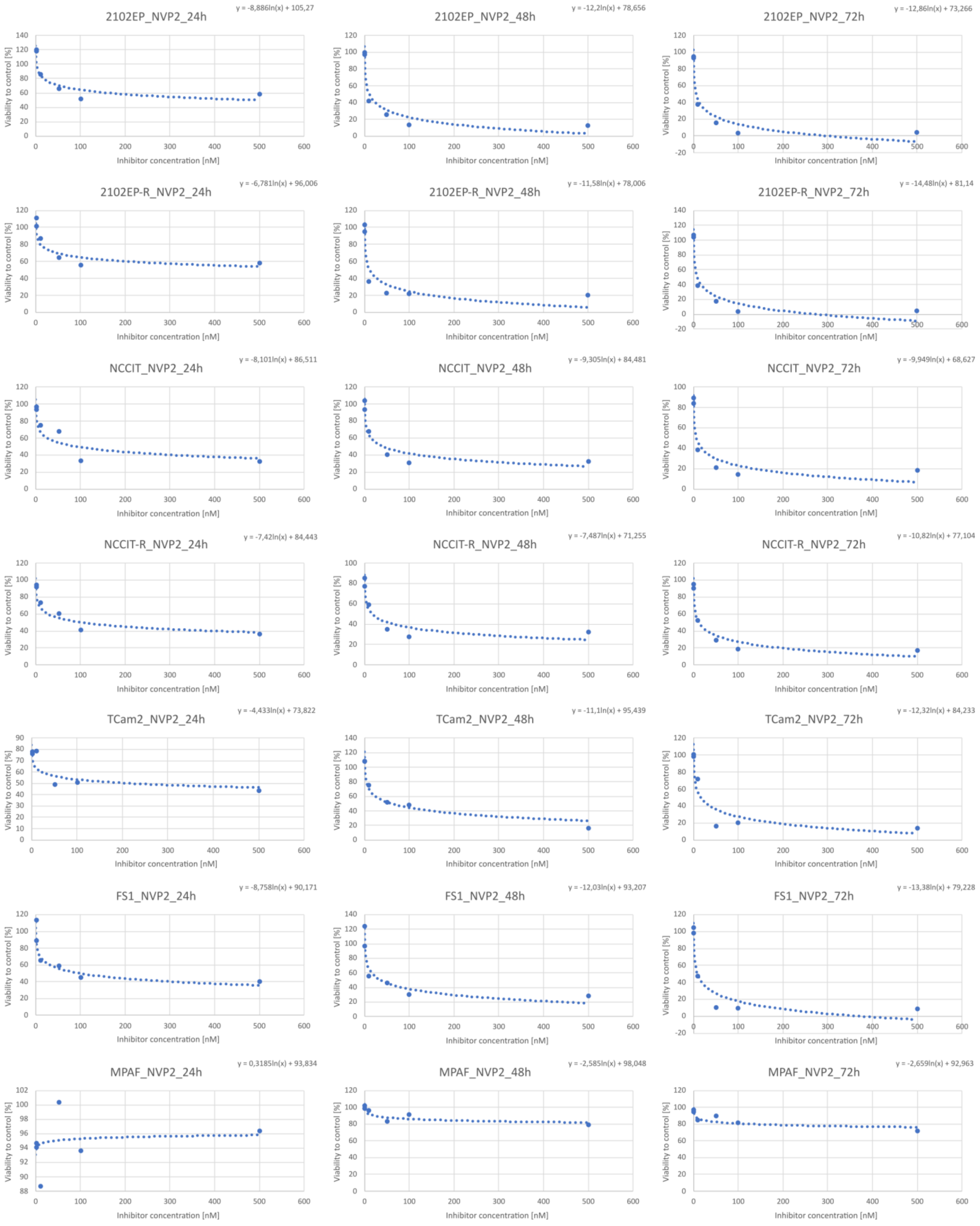

Supplementary Figure S1: Logarithmic regression for NVP2 IC50 value calculation. Graphs are based on XTT viability data from Figure 2.

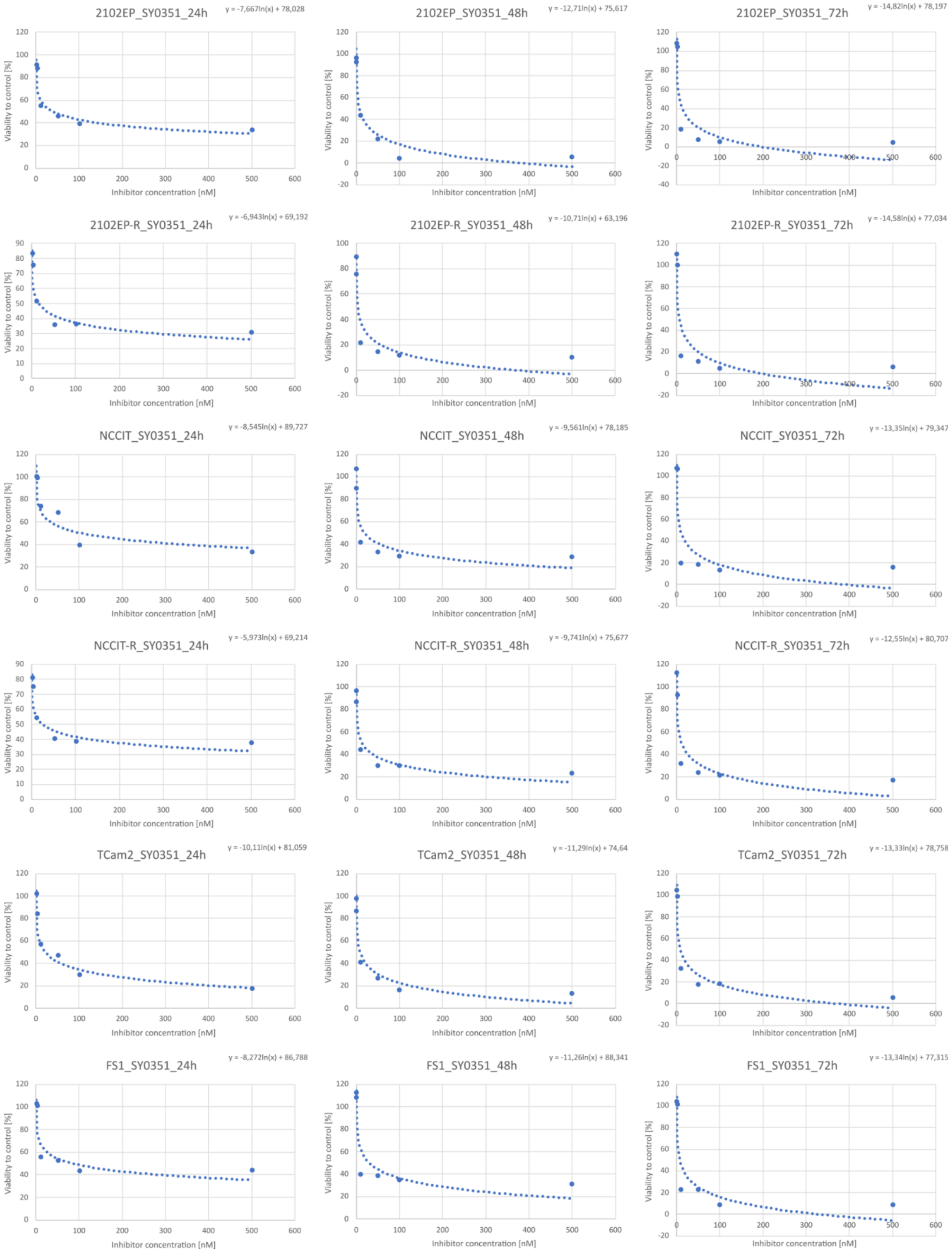

Supplementary Figure S2: Logarithmic regression for SY0351 IC50 value calculation. Graphs are based on XTT viability data from Figure 2.

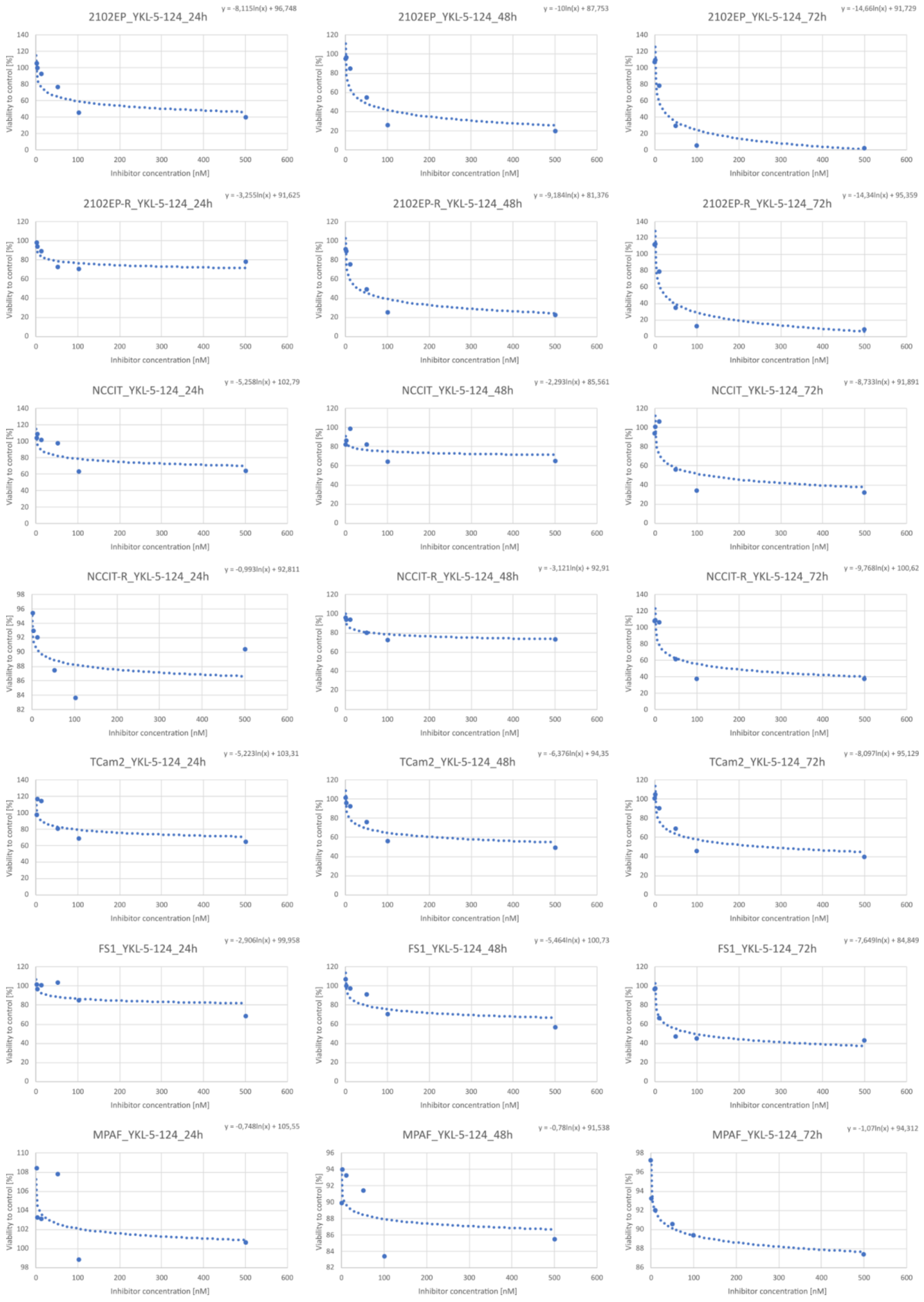

Supplementary Figure S3: Logarithmic regression for YKL-5-124 IC50 value calculation. Graphs are based on XTT viability data from Figure 2.

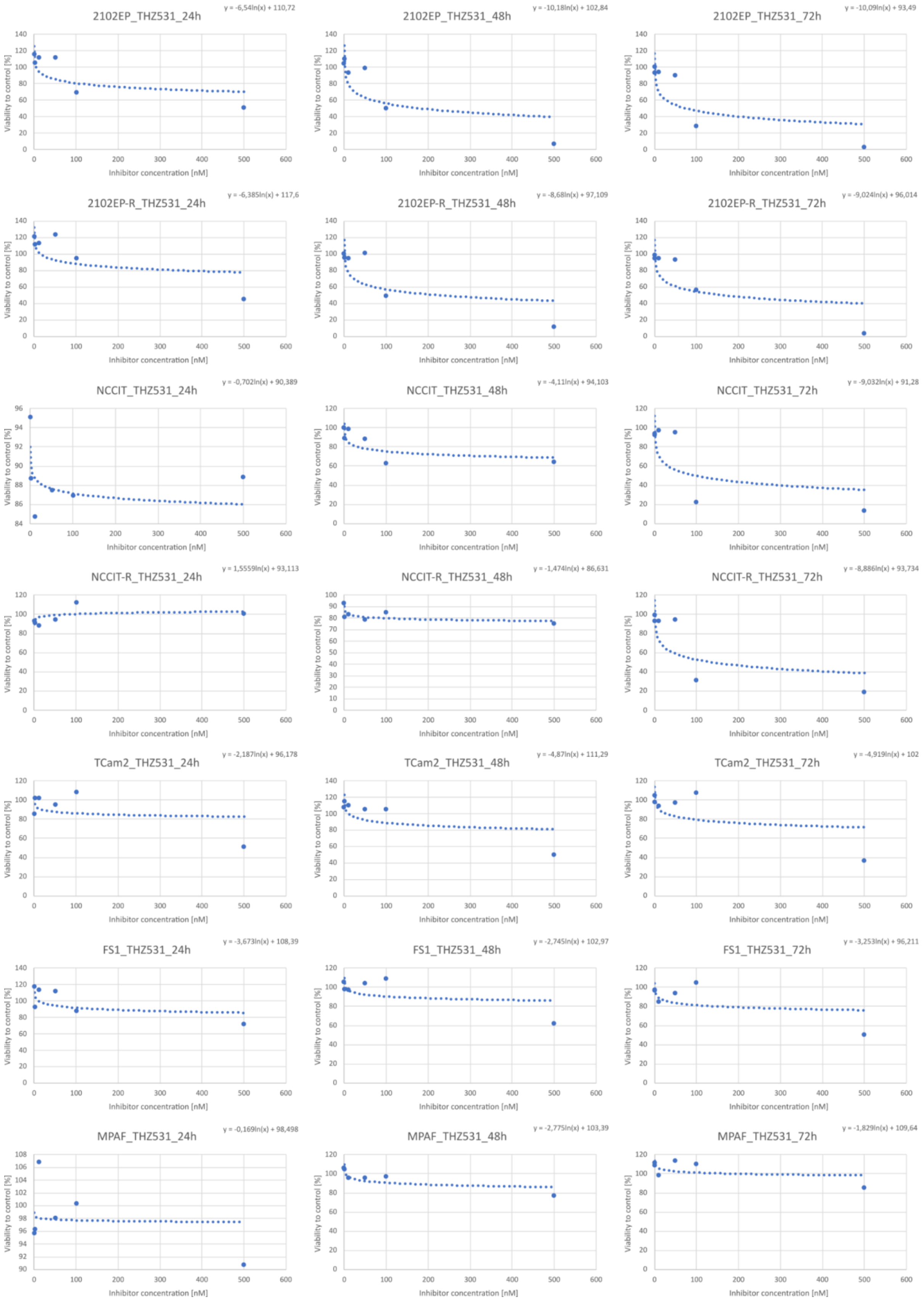

Supplementary Figure S4: Logarithmic regression for THZ531 IC50 value calculation. Graphs are based on XTT viability data from Figure 2.

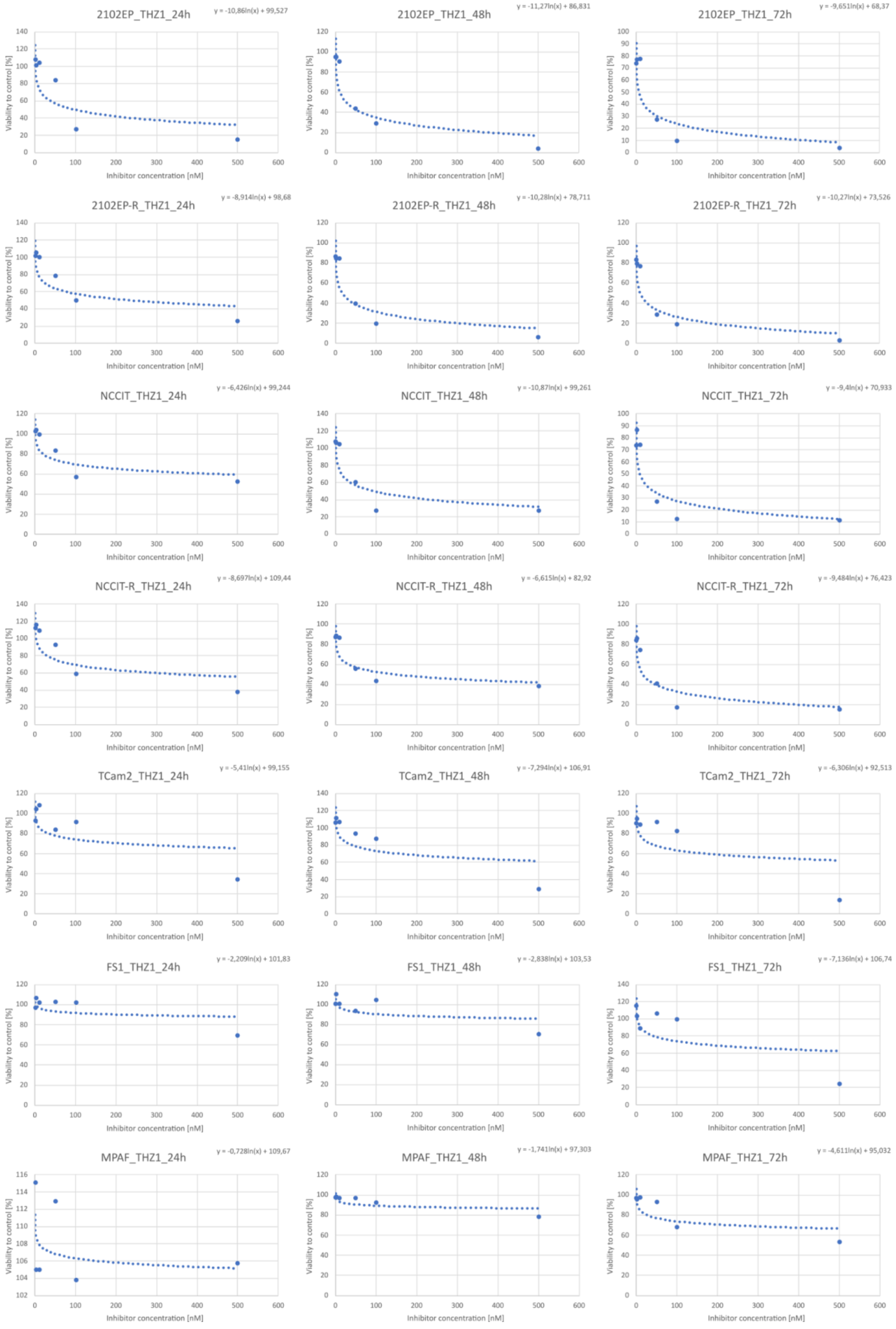

Supplementary Figure S5: Logarithmic regression for THZ1 IC50 value calculation. Graphs are based on XTT viability data from Figure 2.

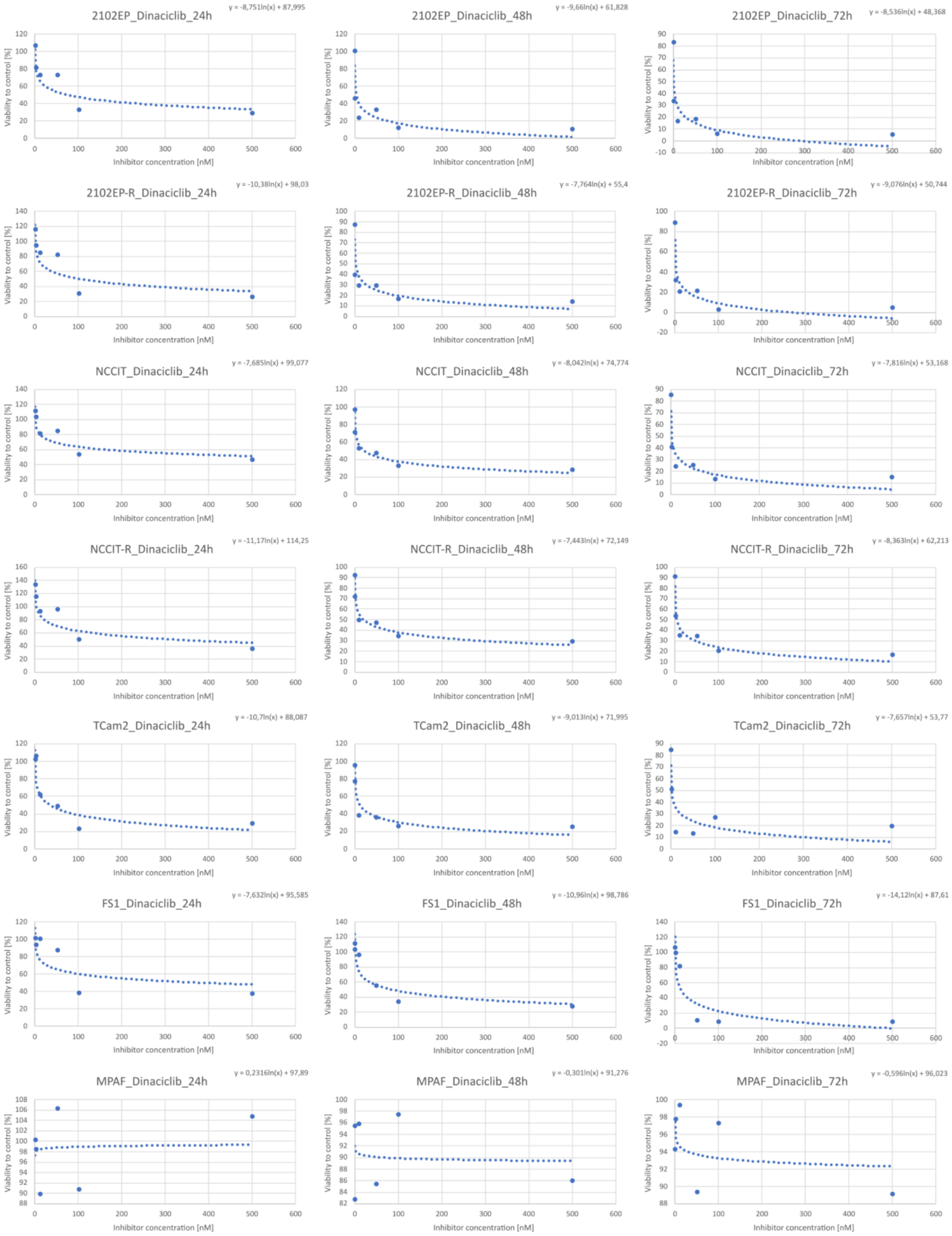

Supplementary Figure S6: Logarithmic regression for Dinaciclib IC50 value calculation. Graphs are based on XTT viability data from Figure 2.

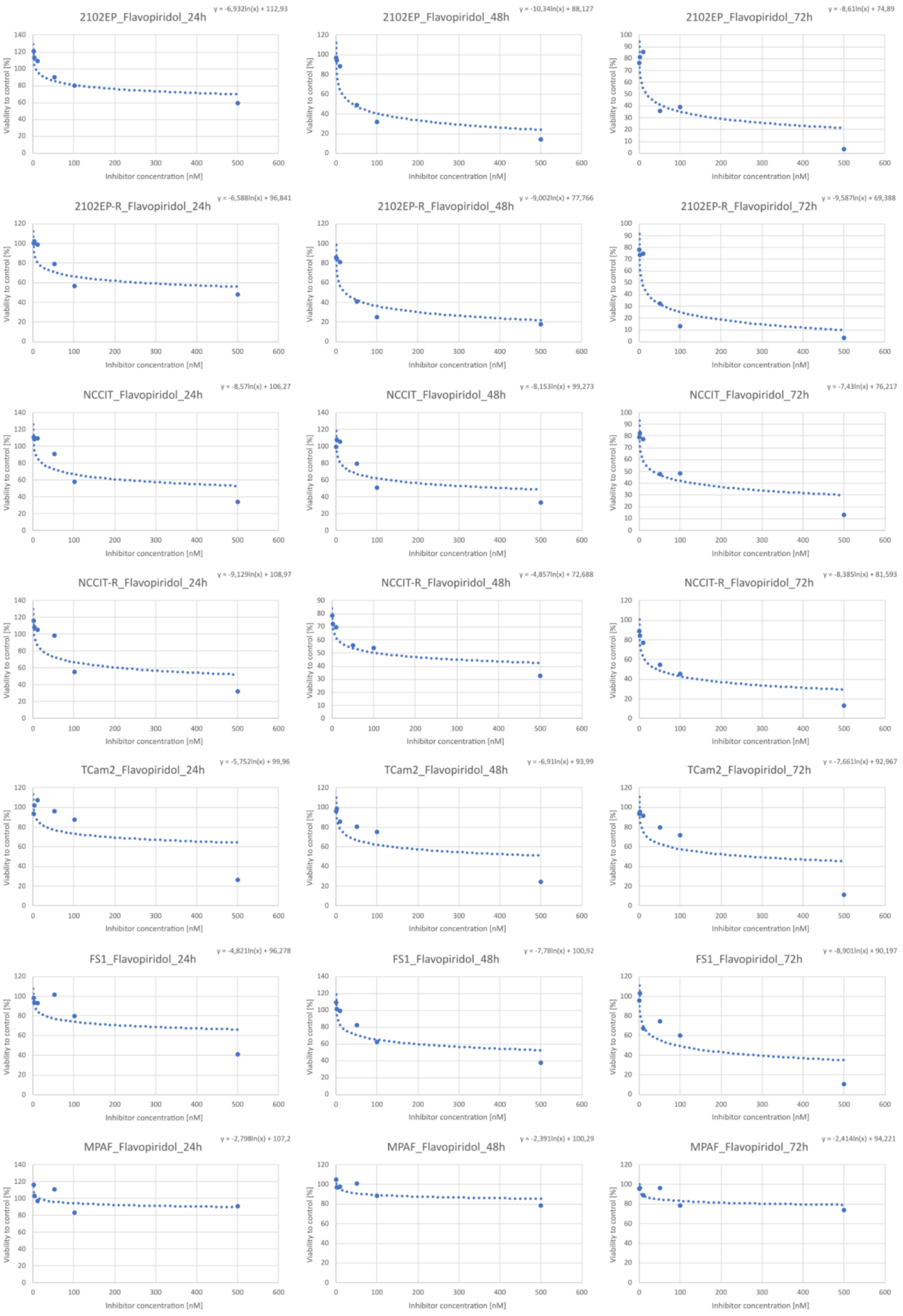

Supplementary Figure S7: Logarithmic regression for Flavopiridol IC50 value calculation. Graphs are based on XTT viability data from Figure 2.

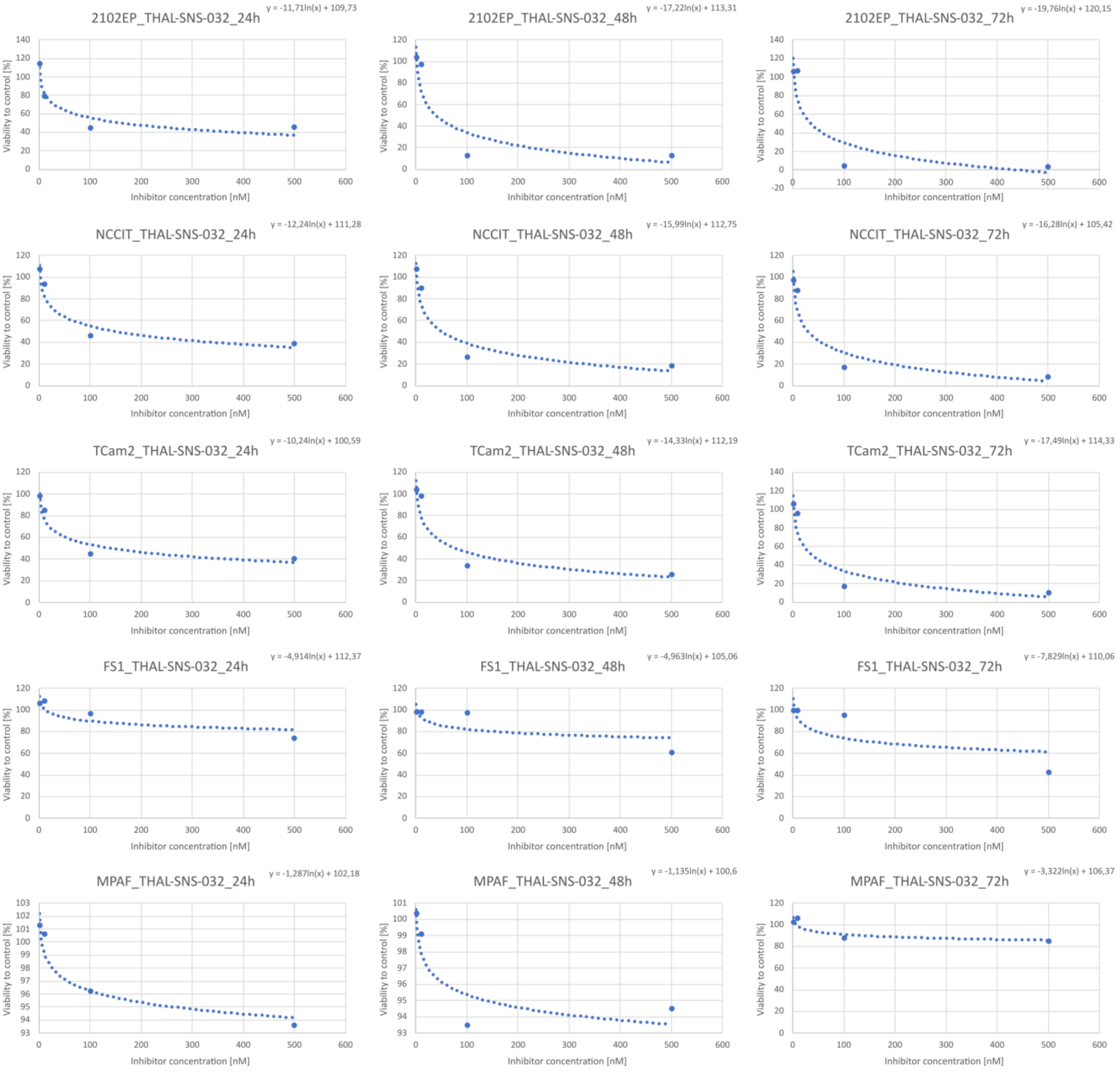

Supplementary Figure S8: Logarithmic regression for THAL-SNS-032 IC50 value calculation. Graphs are based on XTT viability data from Figure 2.

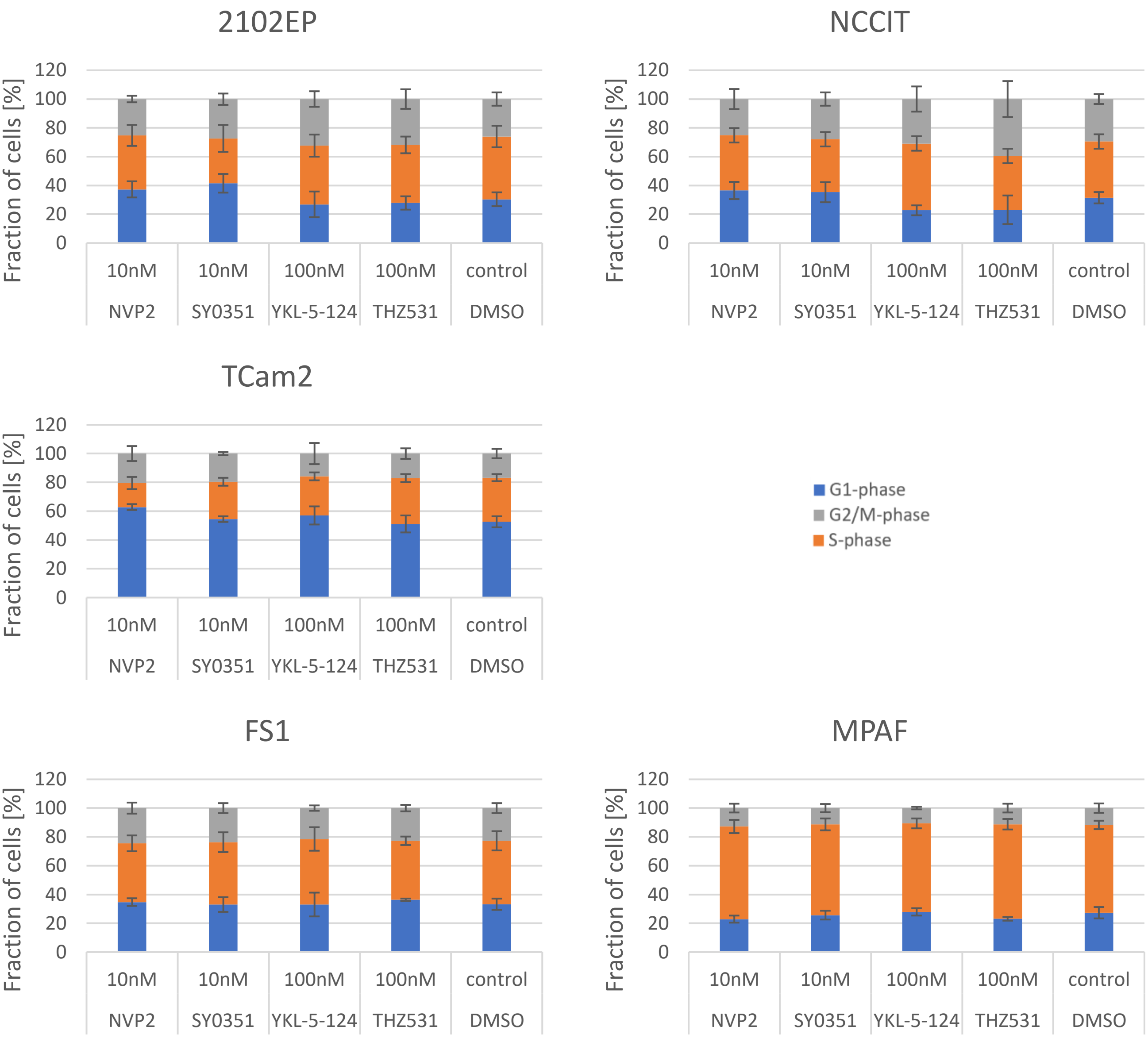

Supplementary figure S9: Cell cycle analysis of TGCT and control cell lines. Hoechst-FACS based cell cycle analysis after 20 h of CKD inhibitor treatment. Control sample was supplemented with DMSO according to inhibitor dilution (Final DMSO conc. 0.0002 %). n=3-6.

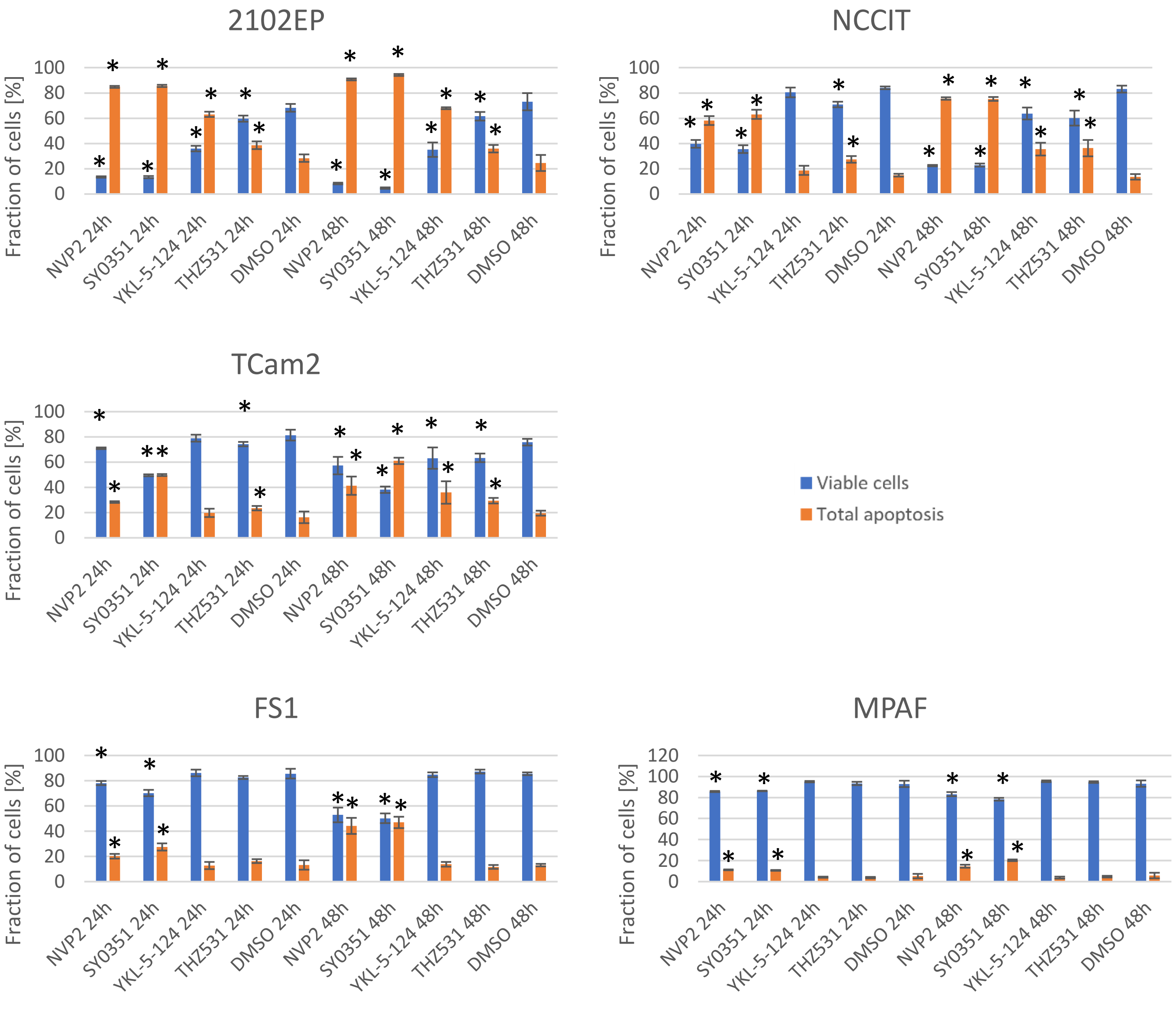

Supplementary figure S10: Apoptosis analysis of TGCT and control cell lines. 7AAD/AnnexinV FACS based apoptosis analysis after 24 h and 48 h of CKD inhibitor treatment. Cells which emitted a signal for AnnexinV only or for Annexin and 7AAD were considered to be apoptotic while double negative cells represented the viable cell population. For calculation of p-values by two-tailed Student's t-test each treated sample is referred to the corresponding DMSO control. n=3-6. Significance is given as *$P < 0.05$.

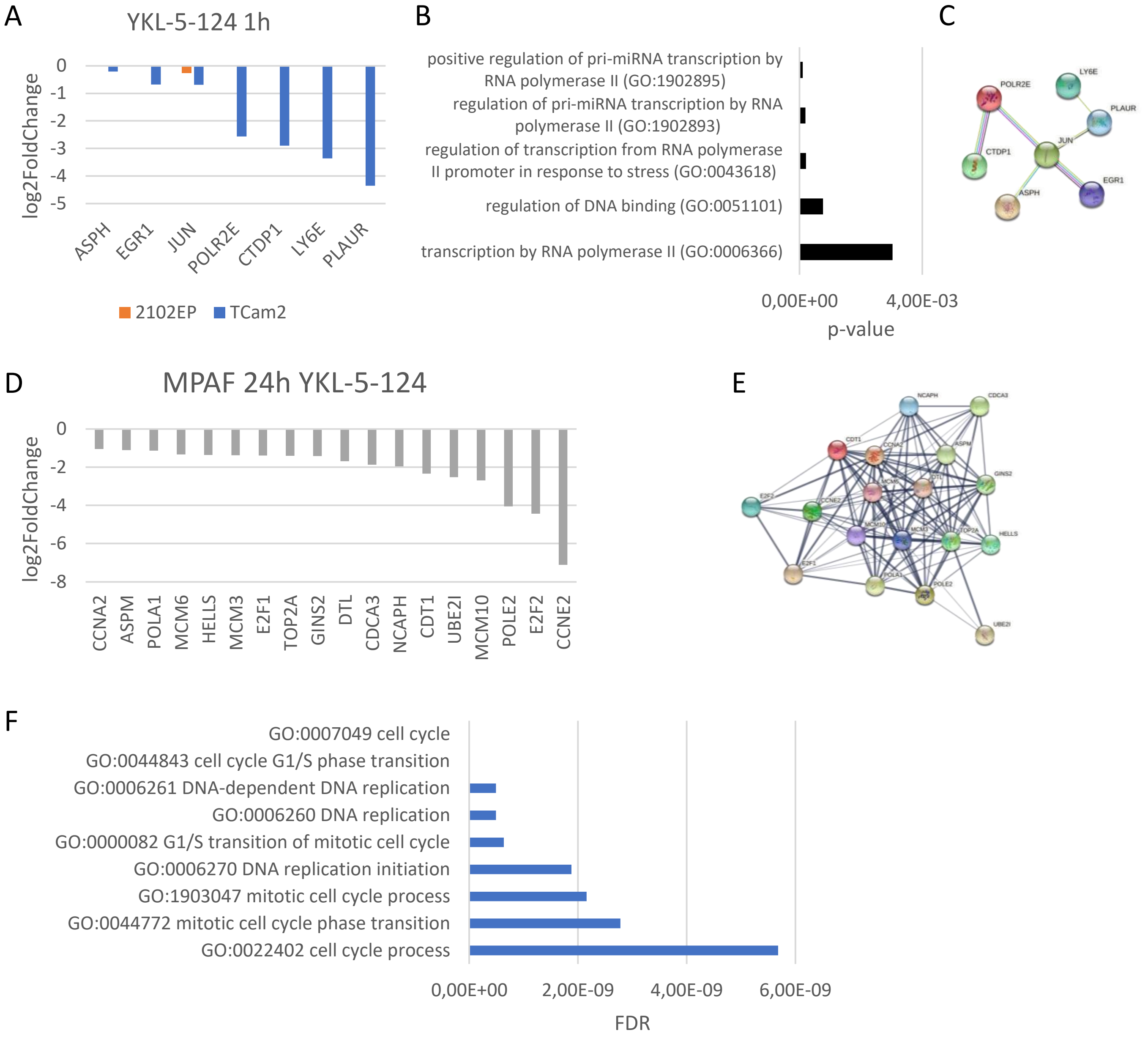

Supplementary figure S11: YKL-5-124 treatment revealed deregulated RNA polymerase II function and downregulation of cell cycle associated genes in TCam2 cells and MPAF cells, respectively. (**A**) Downregulated genes after 1 h of YKL-5-124 (100 nM) treatment in 2102EP and TCam2 cells and corresponsding (**B**) Gene Ontology as well as (**C**) STRING interaction analysis. (**D**) Downregulated genes in MPAF cells after 24 h of YKL-5-124 exposure with (**E**) STRING interaction and (**F**) GO analysis. All samples n=3.

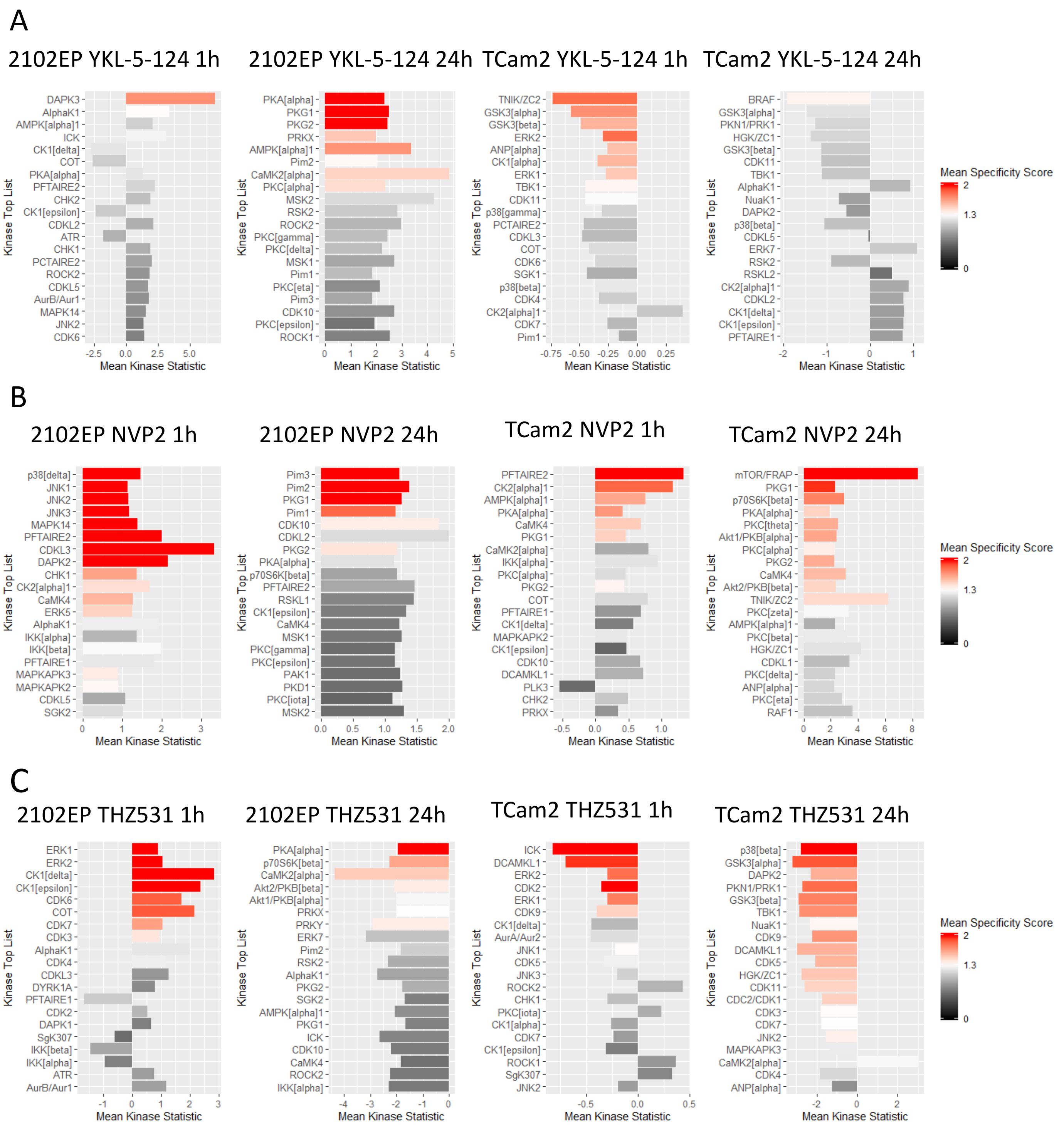

Supplementary Figure S12: Serine/ threonine kinase activity assay. Kinase score plots rank-order the top kinases differential between the treated and DMSO control group. 2102EP and TCam2 cells were treated for 1 h or 24 h with (**A**) YKL-5-124 (100 nM), (**B**) NVP2 (10 nM) or (**C**) THZ531 (100 nM). Subsequently, proteins were isolated and analyzed by PAM Gene assay. All samples n=2.

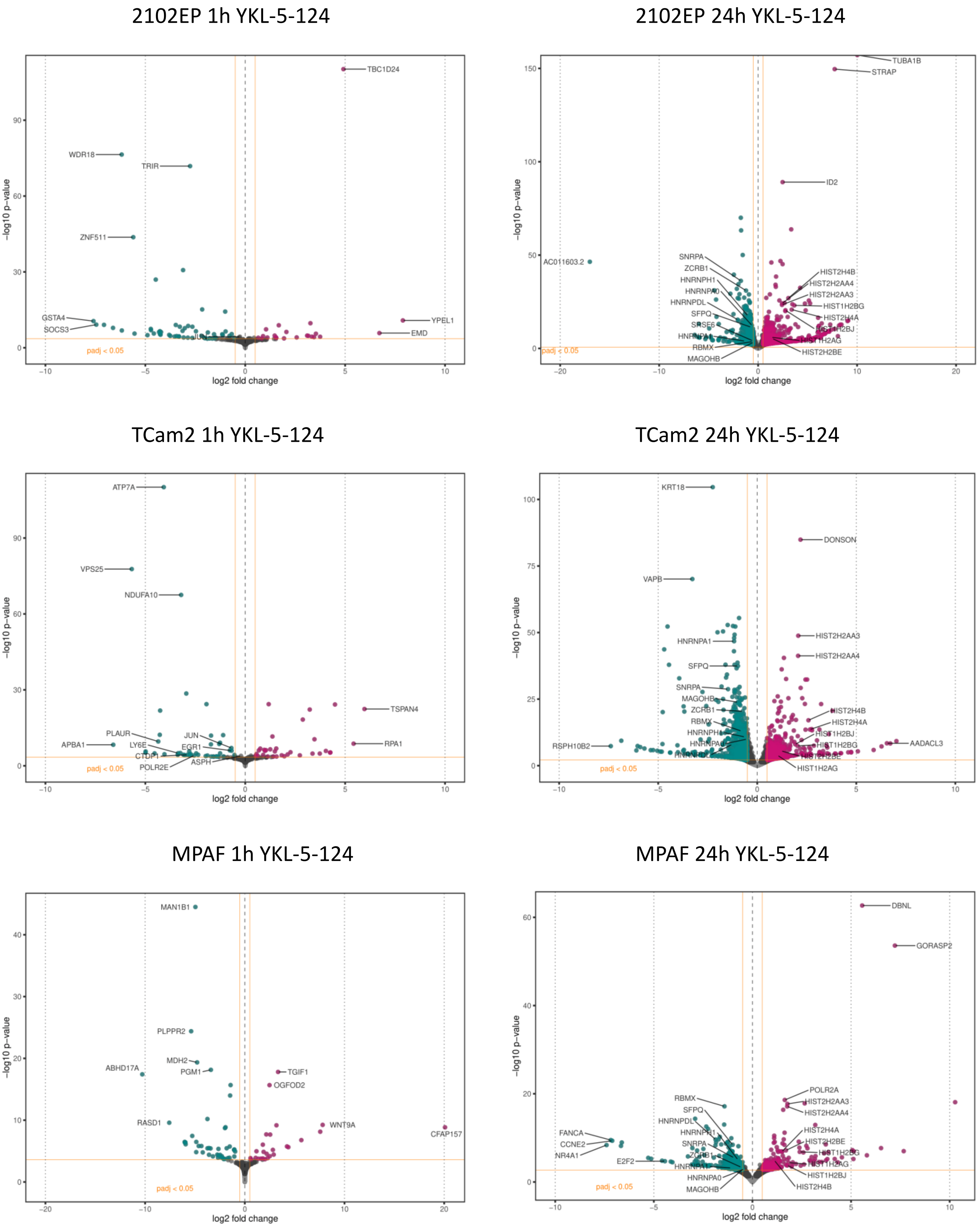

Supplementary Figure S13: RNA sequencing data distribution of significantly deregulated genes (YKL-5-124). 2102EP, TCam2 and MPAF cells were treated for 1 h and 24 h with YKL-5-124 (100 nM).

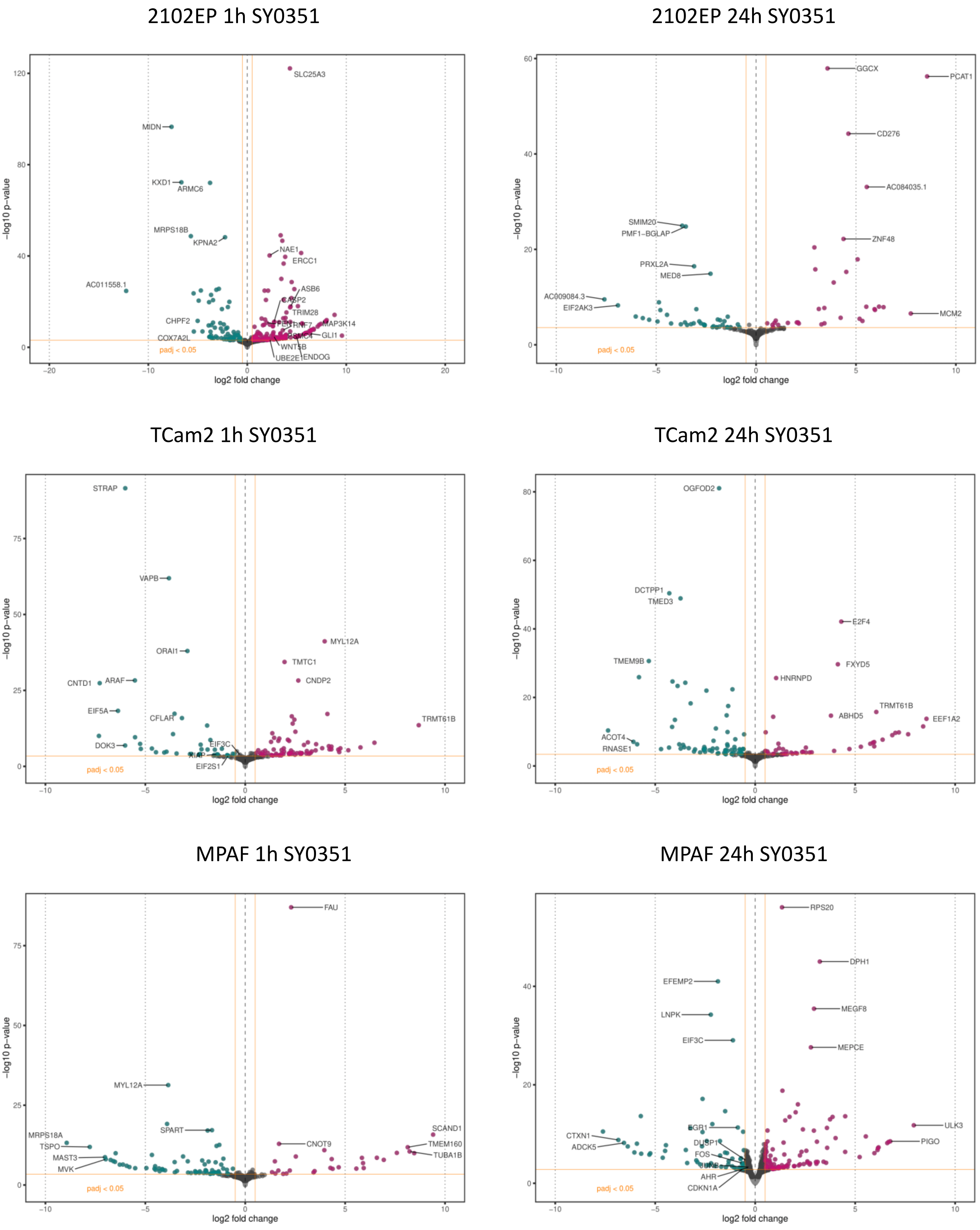

Supplementary Figure S14: RNA sequencing data distribution of significantly deregulated genes (SY0351). 2102EP, TCam2 and MPAF cells were treated for 1 h and 24 h with SY0351 (10 nM).

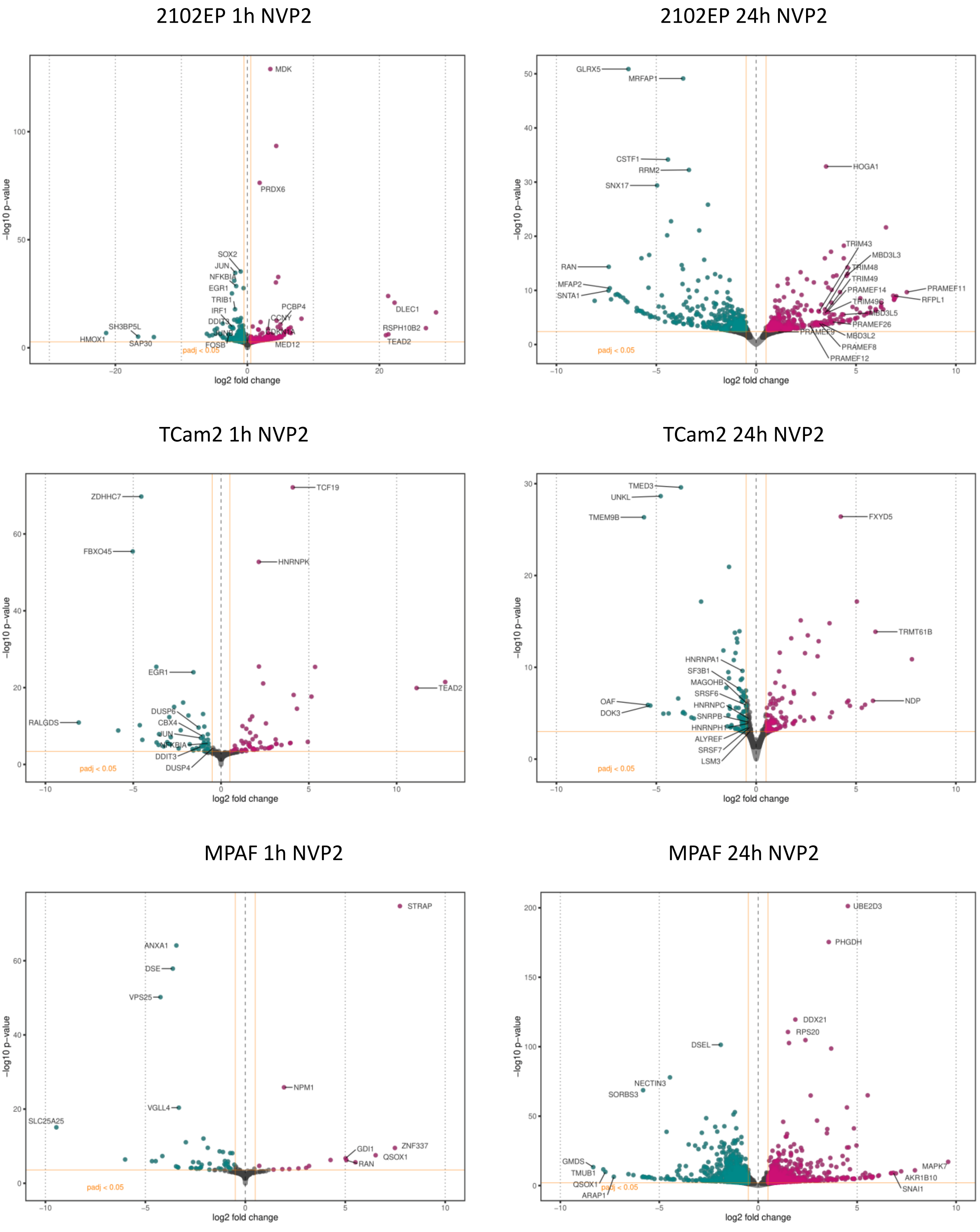

Supplementary Figure S15: RNA sequencing data distribution of significantly deregulated genes (NVP2). 2102EP, TCam2 and MPAF cells were treated for 1 h and 24 h with NVP2 (10 nM).

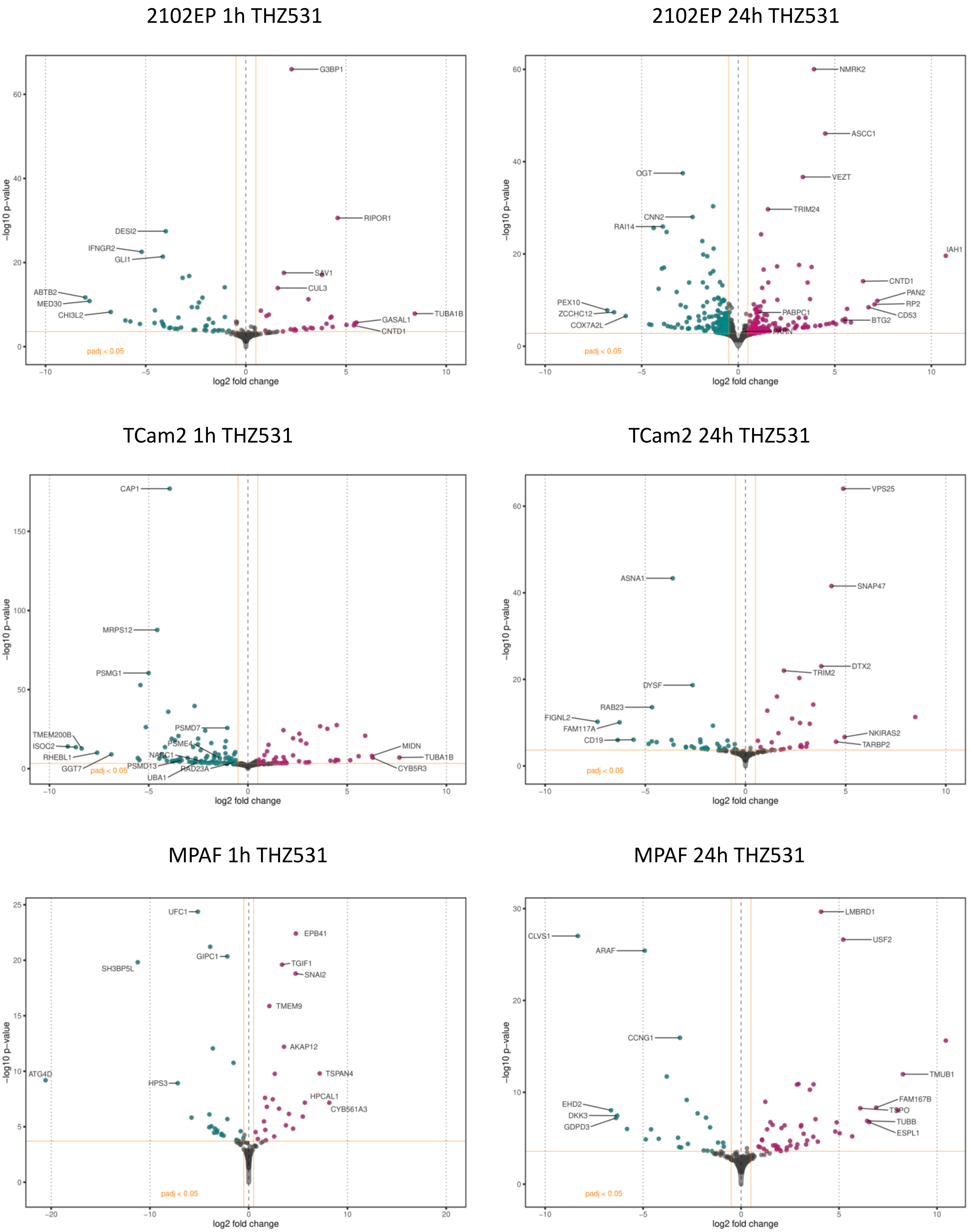

Supplementary Figure S16: RNA sequencing data distribution of significantly deregulated genes (THZ531). 2102EP, TCam2 and MPAF cells were treated for 1 h and 24 h with THZ531 (100 nM).
