# Supplementary figures and images for: Transcriptional CDK Inhibitors as Potential Treatment Option for Testicular Germ Cell Tumors
Source: Cancers (Basel). 2022 Mar 26;14(7):1690. doi: 10.3390/cancers14071690 (PMC8997165; doi:10.3390/cancers14071690)

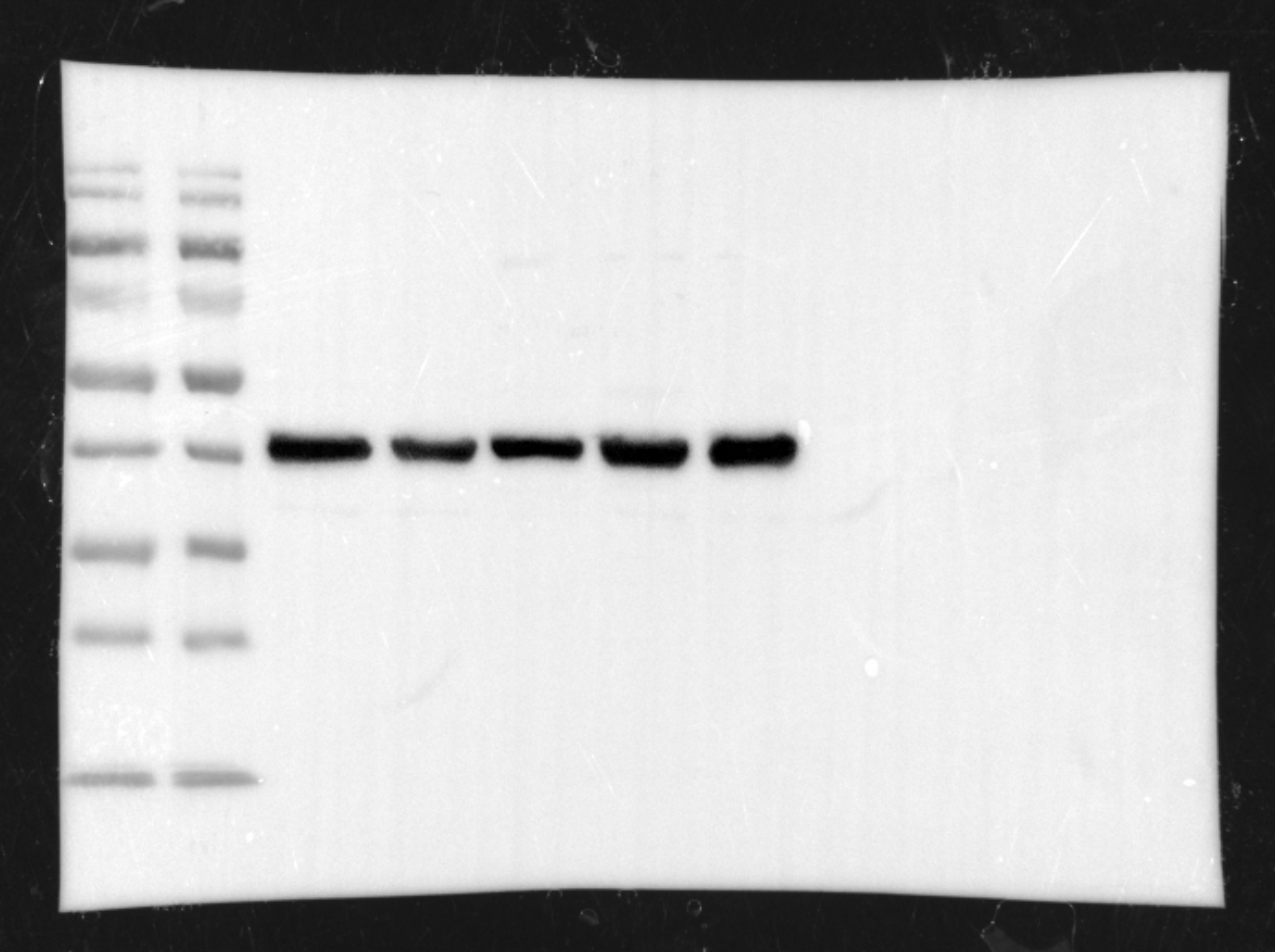

Supplement: Supplementary file 1 [file cancers-14-01690-s001.zip › cancers-1644300 supplementary/1_WB_betaActin_original.tif]

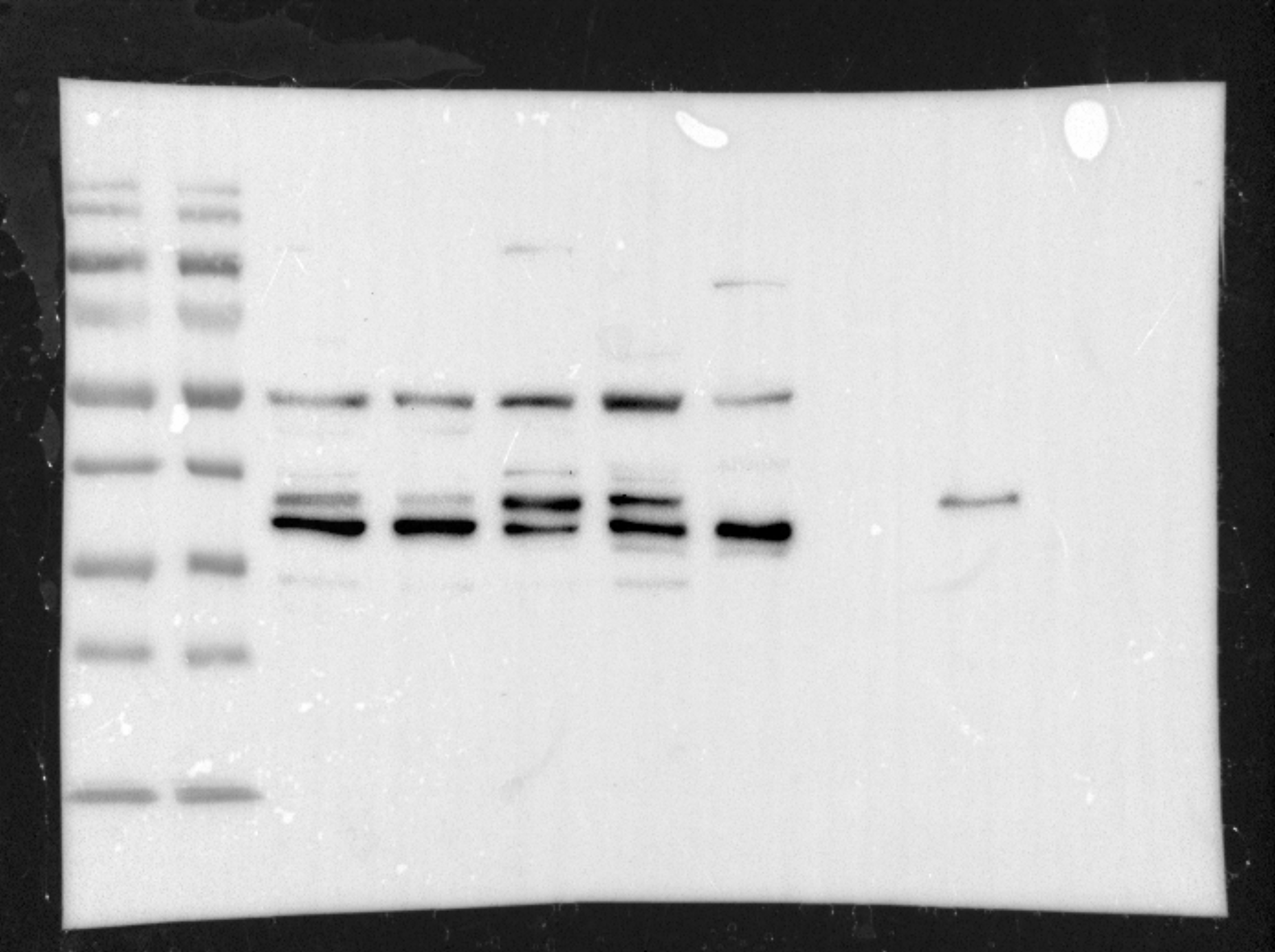

Supplement: Supplementary file 1 [file cancers-14-01690-s001.zip › cancers-1644300 supplementary/1_WB_CDK7_original.tif]

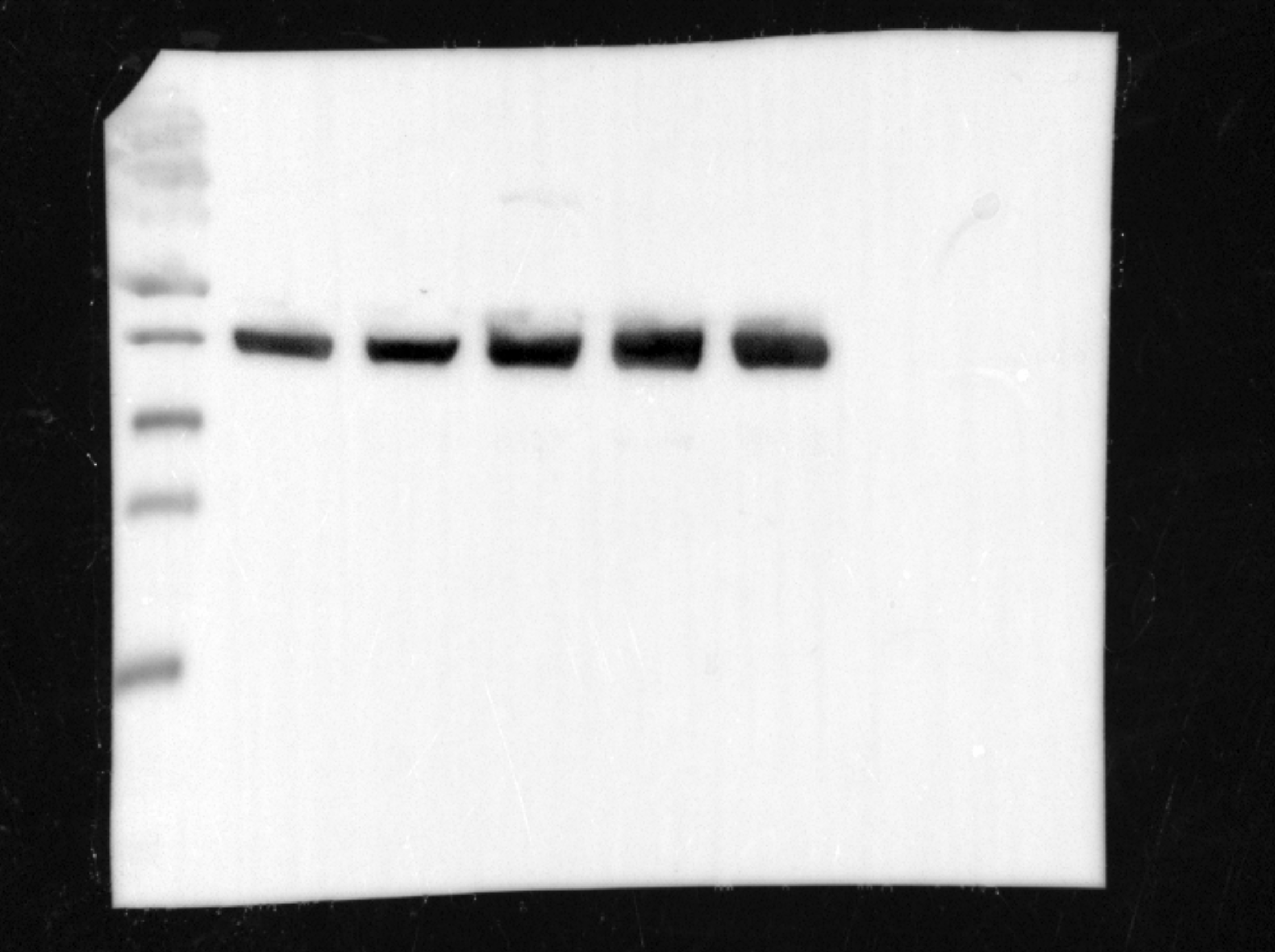

Supplement: Supplementary file 1 [file cancers-14-01690-s001.zip › cancers-1644300 supplementary/2_WB_betaActin_original.tif]

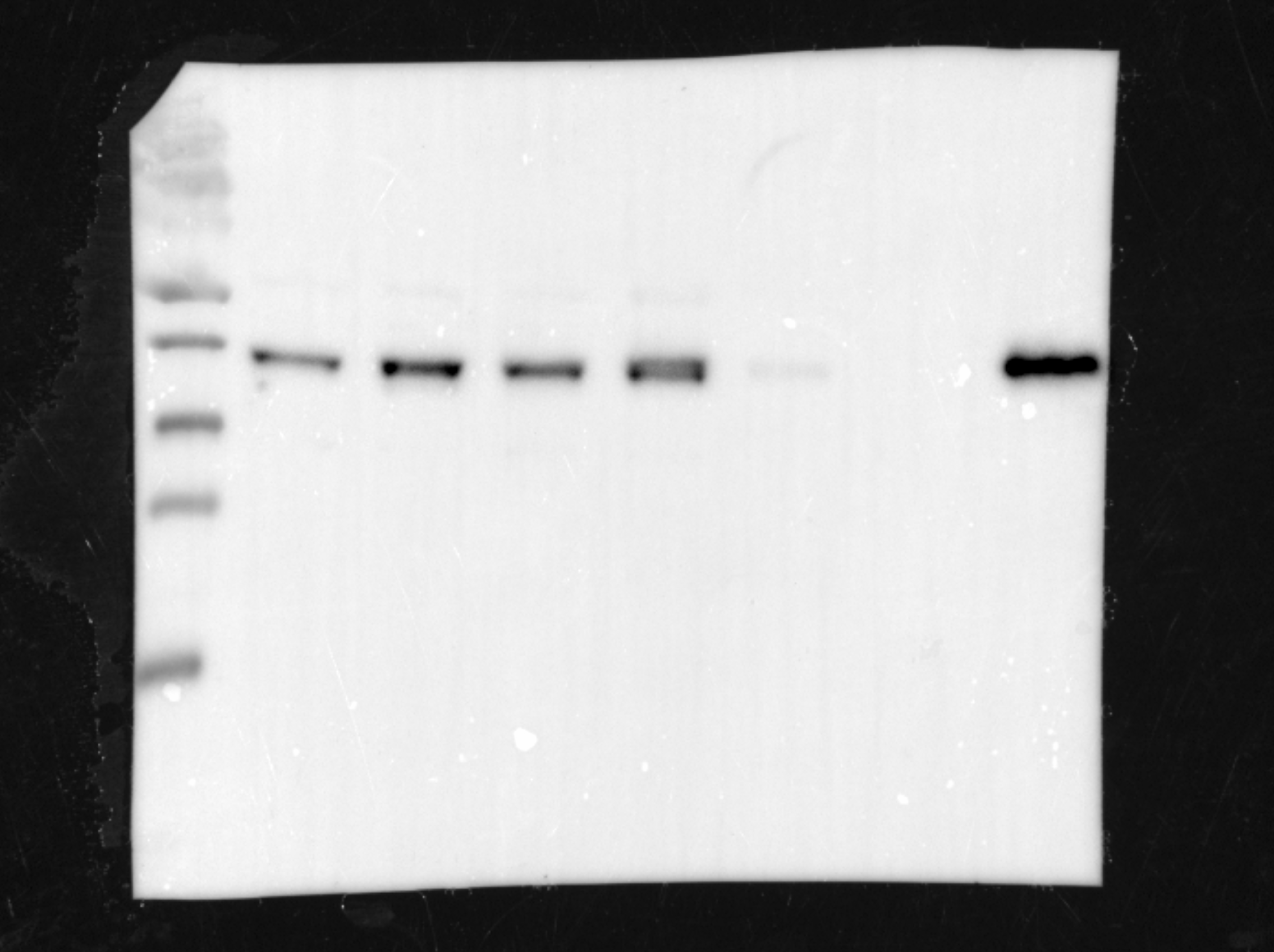

Supplement: Supplementary file 1 [file cancers-14-01690-s001.zip › cancers-1644300 supplementary/2_WB_CDK9_original.tif]

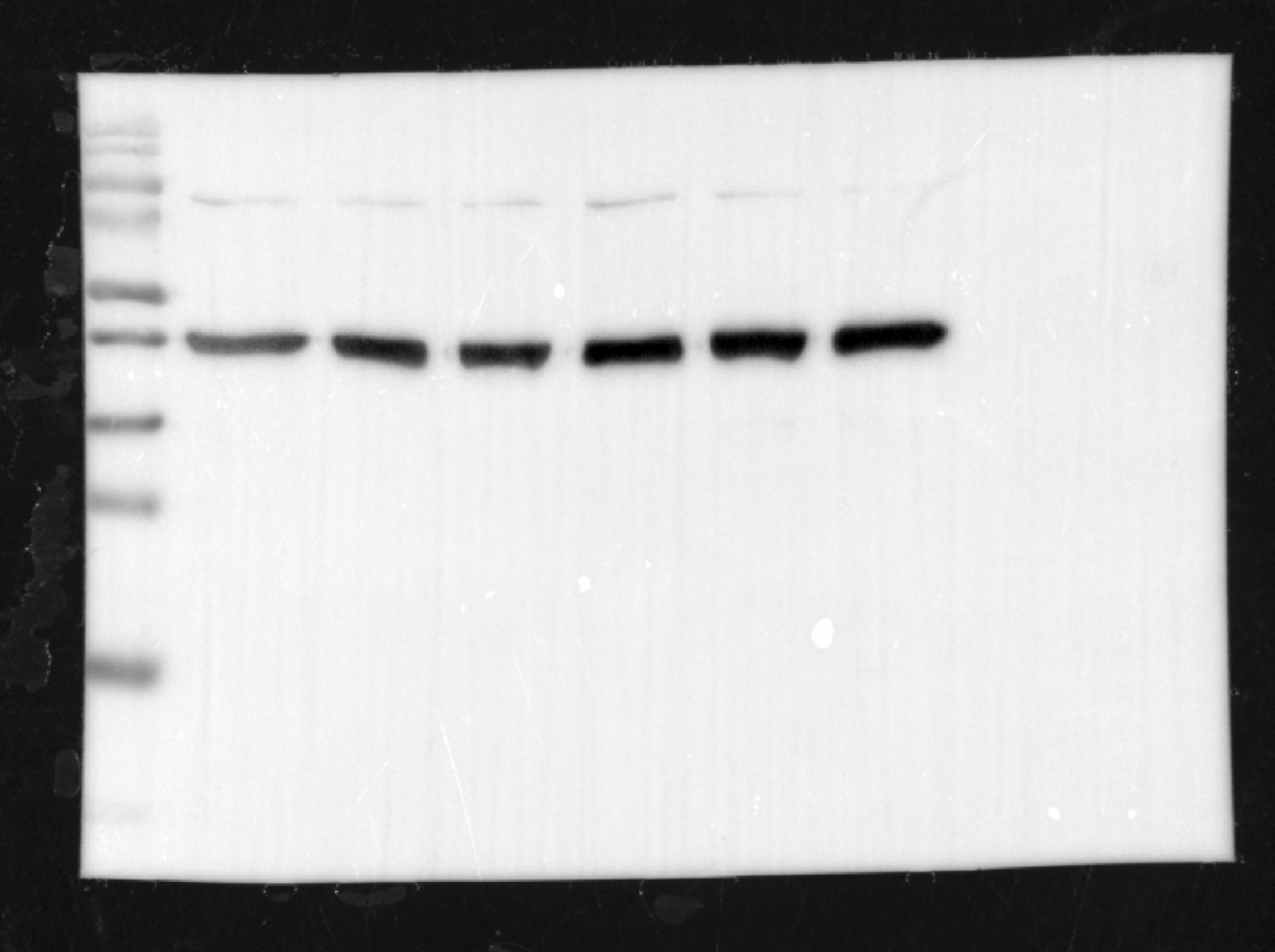

Supplement: Supplementary file 1 [file cancers-14-01690-s001.zip › cancers-1644300 supplementary/3_betaActin_original.tif]

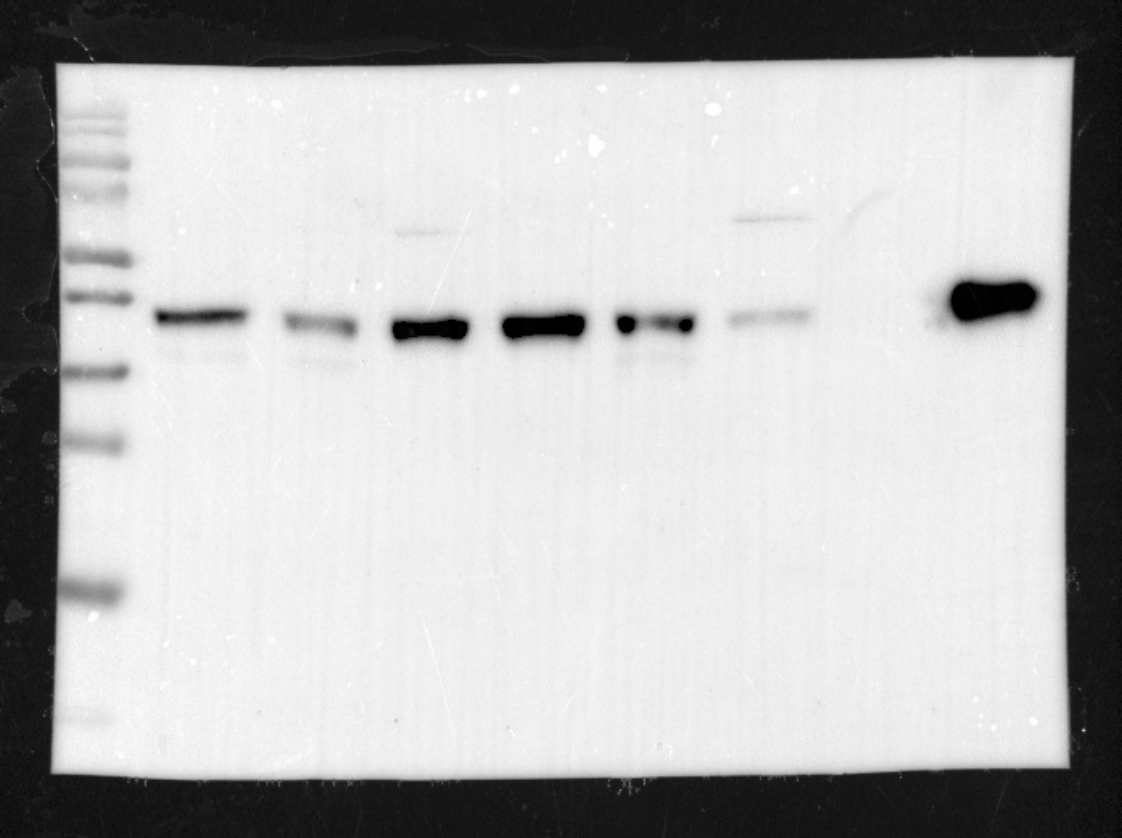

Supplement: Supplementary file 1 [file cancers-14-01690-s001.zip › cancers-1644300 supplementary/3_CDK10_original.tif]

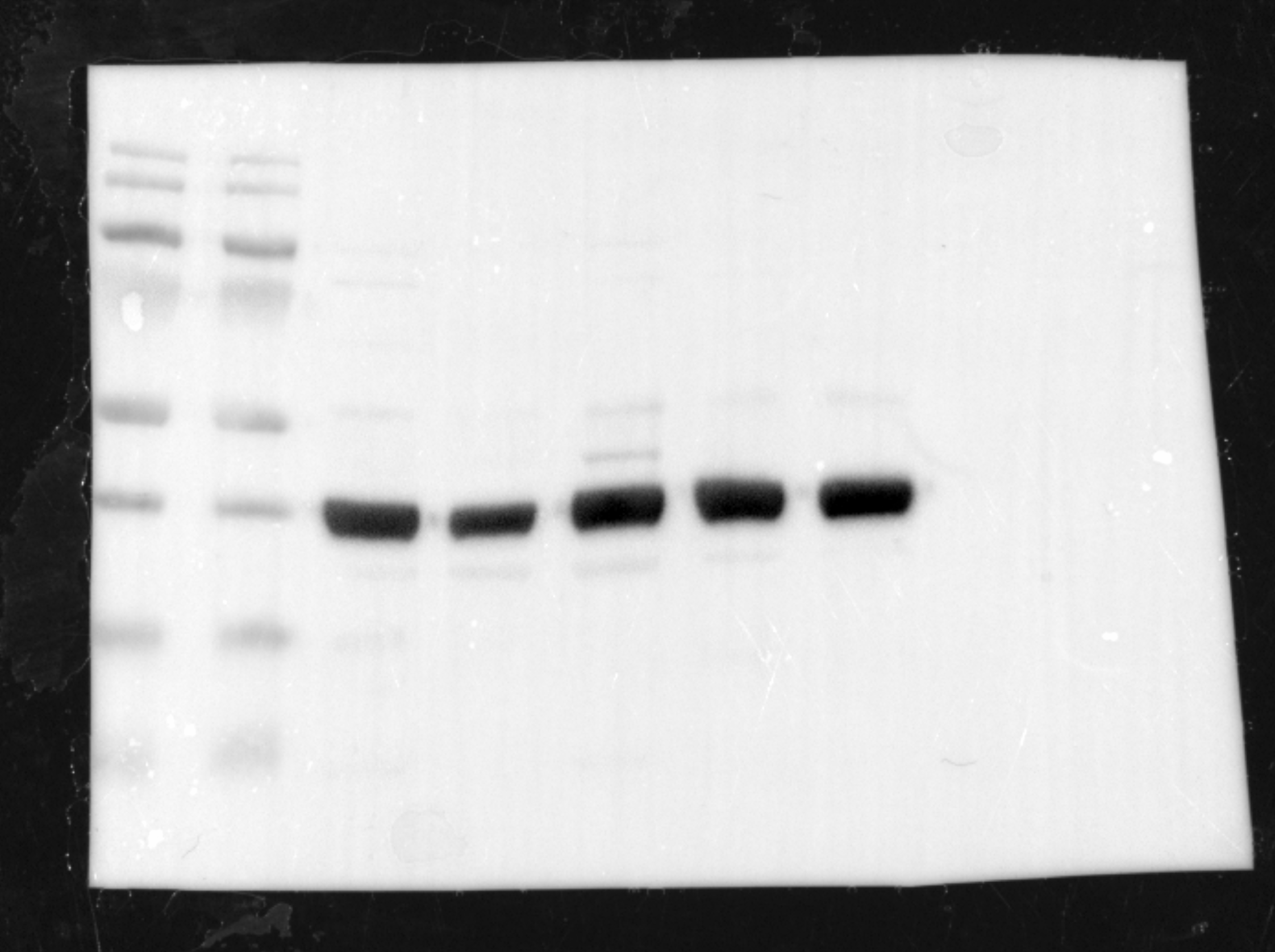

Supplement: Supplementary file 1 [file cancers-14-01690-s001.zip › cancers-1644300 supplementary/4_WB_betaActin_original.tif]

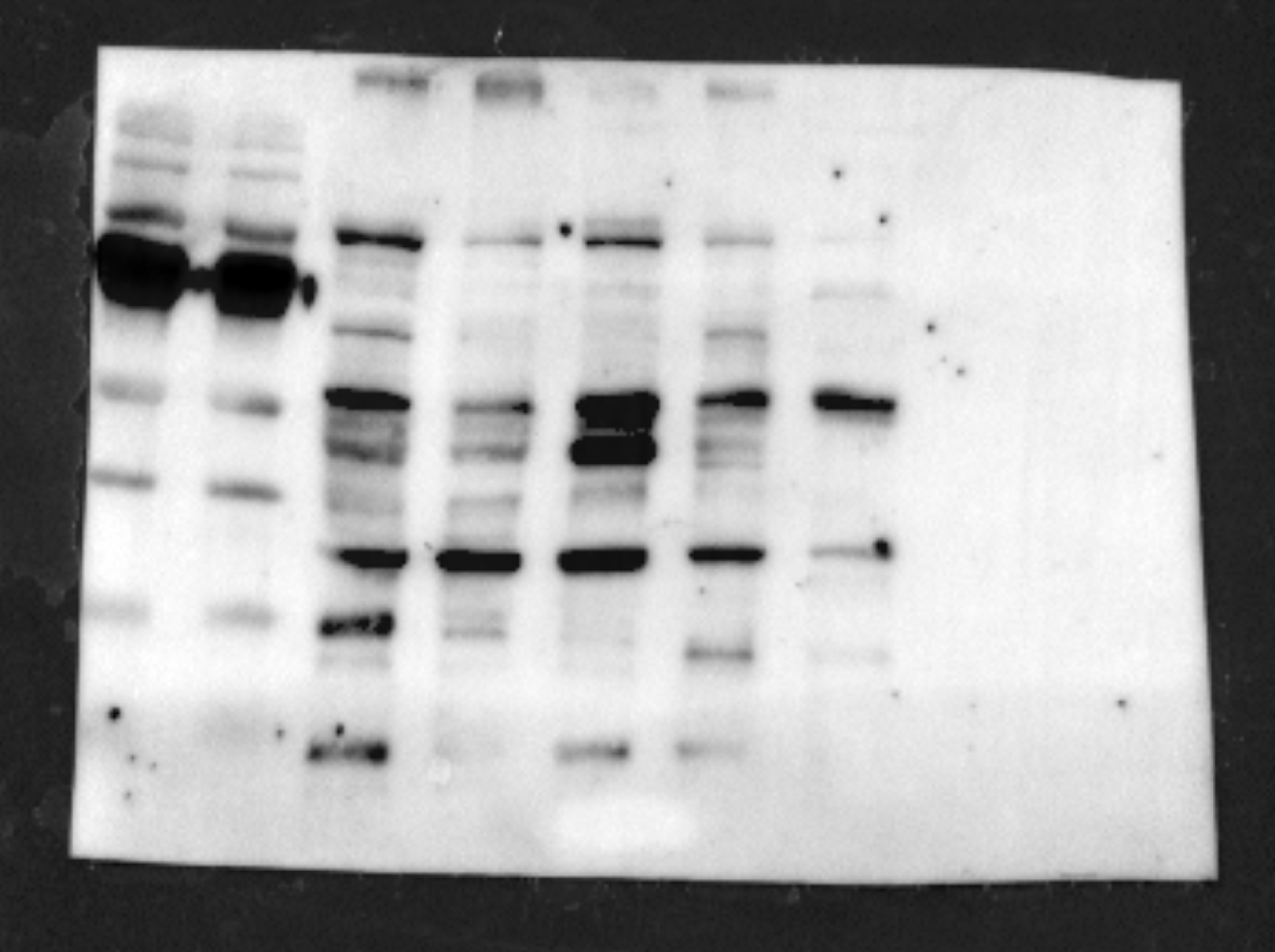

Supplement: Supplementary file 1 [file cancers-14-01690-s001.zip › cancers-1644300 supplementary/4_WB_CDK12_original.tif]

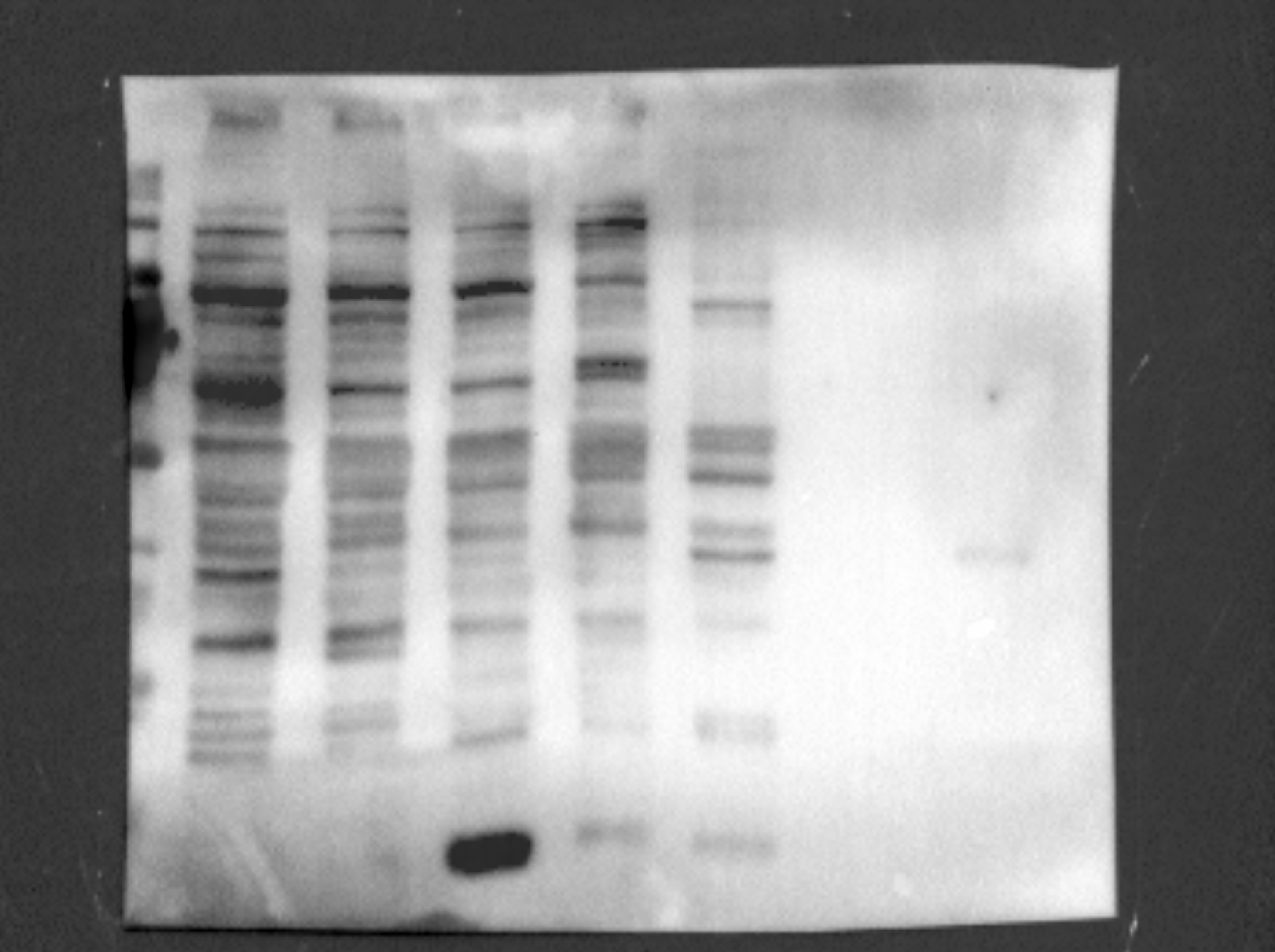

Supplement: Supplementary file 1 [file cancers-14-01690-s001.zip › cancers-1644300 supplementary/5_CDK13_original.tif]

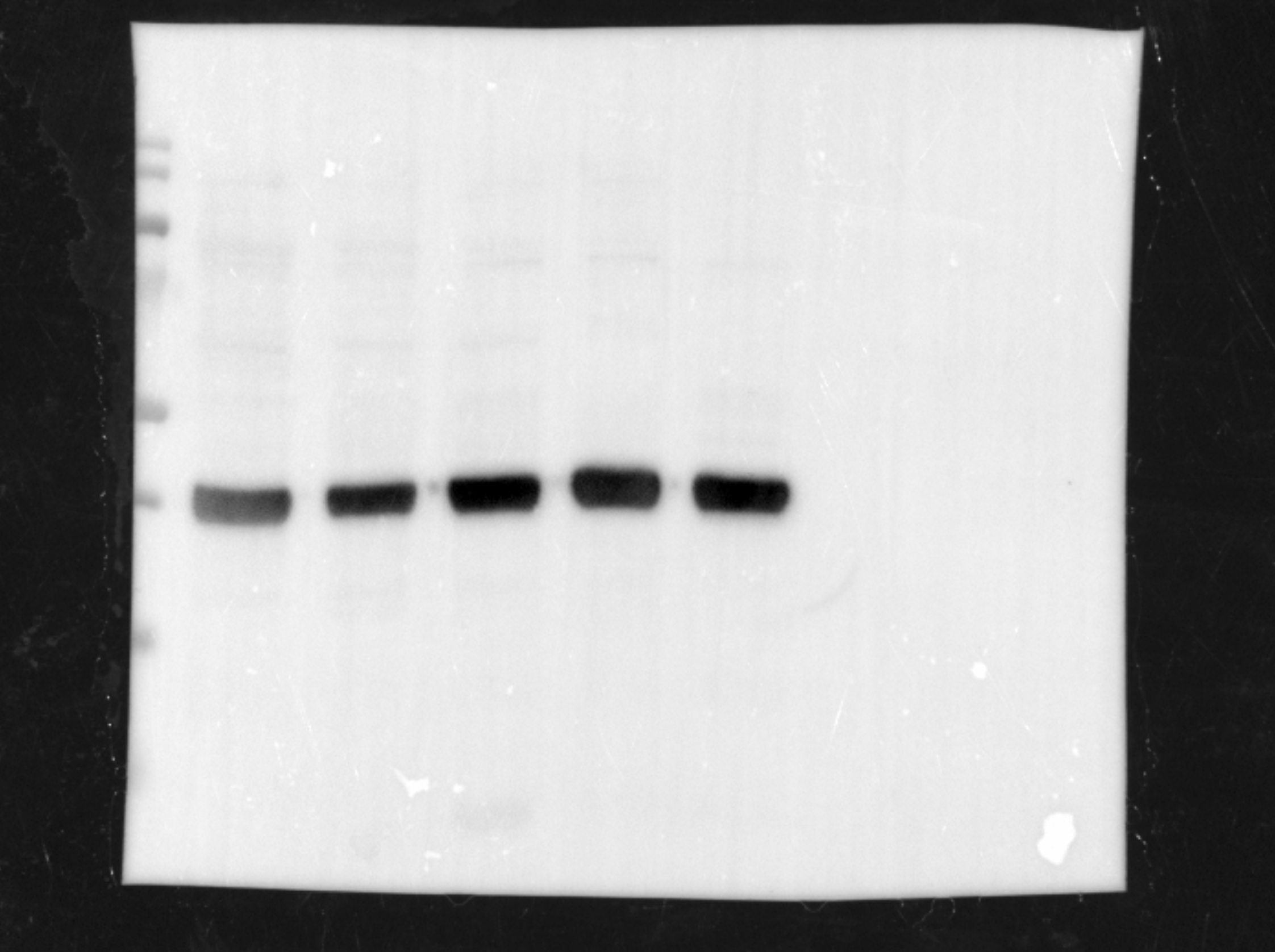

Supplement: Supplementary file 1 [file cancers-14-01690-s001.zip › cancers-1644300 supplementary/5_WB_betaActin_original.tif]
